# Industrial diet intervention modulates the interplay between gut microbiota and host in semi-stray dogs

**DOI:** 10.1186/s42523-024-00357-w

**Published:** 2024-11-21

**Authors:** Soufien Rhimi, Amin Jablaoui, Juan Hernandez, Vincent Mariaule, Nizar Akermi, Tristan Méric, Héla Mkaouar, Magdalena Wysocka, Adam Lesner, Mohamed Ali Borgi, Emmanuelle Maguin, Moez Rhimi

**Affiliations:** 1 Microbiota Interaction with Human and Animal Team (MIHA), Micalis Institute, Institut National de Recherche Pour l’Agriculture, l’Alimentation et l’Environnement, AgroParisTech, Université Paris-Saclay, 78350 Jouy-en-Josas, France; 2 Oniris VetAgroBio Nantes, Department of Clinical Sciences, Nantes-Atlantic College of Veterinary Medicine and Food Sciences, 44300 Nantes, France; 3 Faculty of Chemistry, University of Gdansk, Wita Stwosza 63, 80308 Gdańsk, Poland; 4 Laboratory of Biotechnology and Biomonitoring of the Environment and Oasis Ecosystems (LBBEEO), Faculty of Sciences of Gafsa, University of Gafsa, Zarroug, 2112 Gafsa, Tunisia

**Keywords:** Dog, Diet intervention, Semi-stray dog, Industrial diet, Gut microbiota, Host response, Holobiont

## Abstract

**Background:**

The gut microbiota and derived metabolites play a key role in regulating host physiology. Diet is identified as a key regulatory factor of the microbiota composition and, potentially, of subsequent functionalities. Demonstrating the role of diet may be complex as most human studies are cross-sectional and dietary intervention is often accompanied by hygienic changes. The objective of the present study was to investigate the impact of an industrial diet on the modulation of the microbiota and targeted functionalities using a canine “natural” model.

**Results:**

We carried out a controlled dietary trial in a cohort of Tunisian semi-stray dogs. We made a transition from a natural diet to an industrial kibble diet and monitored the composition of the fecal microbiota, the concentration of short-chain fatty acids (SCFA) and bile acids (BAs), and protease activities. We demonstrated that dietary change significantly decreased fecal primary bile acids levels and protease activities. Interestingly, correlation analyses demonstrated that variation of specific microbial genera were associated with modulated physiological parameters.

**Conclusions:**

Our study reveals that an industrial diet induces beneficial changes in microbial composition and functions characterised by increased diversity, synthesis of SCFA and secondary bile acids production, stressing the key role of the diet-microbiota-dog crosstalk.

**Supplementary Information:**

The online version contains supplementary material available at 10.1186/s42523-024-00357-w.

## Background

The role of the intestinal microbiota in health is now well-established in humans and animals. Many environmental, medicinal, hygienic, or dietary factors can modify its composition and function and thus have an impact on health and well-being. In humans, the Mediterranean diet (MD) has long been known for its protective effects on cardiovascular health and, more recently, cancer and cognitive health [1, 2]. Typically, MD is a custom food in Latin European Countries, Greece, and North Africa, rich in fruits, vegetables, whole grains, nuts, seeds, onion, garlic, and aromatic herbs. The source of fat is essentially olive oil. Proteins of animal origin are consumed in small proportions [2]. In contrast, the diet of Western industrialised countries (WD) is characterised by the abundant consumption of ultra-processed products rich in refined sugars, animal fat, and poor in fiber [3]. The advent of WD in industrialised countries is associated with an increase in the incidence of chronic noncommunicable diseases (cardiovascular diseases, dyslipidemia, obesity, diabetes mellitus, chronic inflammatory diseases, cancers, and neurodegenerative diseases) [3].

The role of diet in the composition and functions of the intestinal microbiota is well demonstrated within human cohort studies. Greater biodiversity and a relative predominance of *Prevotella*, *Clostridium* of cluster XIVa and *Faecalibacterium prausnitzii* are observed in subjects fed with MD while *Bacteroidetes* are dominant in individuals fed with WD [4]. Among the most important gut microbiota bio-products, short-chain fatty acids (SCFA) and bile acids (BAs) are known to modulate homeostasis. SCFA are produced mainly from the fermentation of non-digestible dietary fiber and starch. SFCA impact the immune and metabolic homeostasis and their concentrations are associated with consuming plant-based nutrients [5, 6]. Bile acids are other microbial bio-metabolites that have been shown to play a key role in intestinal inflammation and host metabolism. Patients with inflammatory bowel disease (IBD) present increased fecal levels of primary conjugated BAs, whereas secondary BAs decrease dramatically compared with healthy subjects [7, 8]. More recently, fecal proteolytic activities have emerged as an important player in the dialogue between the microbiota and the host [9]. Increased serine protease (SP) activity and decrease of their inhibitor, so called serpins, are observed in human and canine patients with IBDs is likely to interact with various signalling pathways, inflict direct tissue damage, and exacerbate gut inflammation [9, 10]. As large number of SP and serpins are encoded by the gut microbiota, determining the role of diet in the modulation of the microbiota and the subsequent regulation of microbial functions involved in gut homeostasis is a future challenge [11]. Most studies on the role of diet in modulating the microbiota and its functions are cross-sectional, meaning they describe the characteristics of cohorts of subjects who adhere to one or the other diet [12]. Interventional studies are rare and dietary changes are associated with other environmental or hygienic (*i.e.*, physical activity) modifications that prevent establishing cause-effect relationships.

The domestic dog is a species of interest because it shares its owners’ living environment and eating habits and can easily be subjected to dietary changes. Similarly to the main types of diets described in humans (MD vs WD), two main trends emerge in animal health. On one hand, a so-called “natural diet” made from fresh, unprocessed products, and on the other hand, an industrial diet mainly composed of kibble. The “natural diet” is composed of fresh products that are not or minimally processed in proportions that are often unbalanced compared to the dog's nutritional recommendations. Kibbles are marketed by large companies specialising in animal feed and are nutritionally balanced. The industrial process of manufacturing kibble requires the presence of a minimum quantity of starch to enable the extrusion process. The semi-stray dogs from Tunisia have the advantage of feeding on “natural products” in a rural setting in a developing country. Semi-wandering allows a controlled dietary trial making it possible to study the effects of diet on the gut microbiota in a complex and stable environment.

Our study aimed to evaluate the effects of the transition from a “natural diet” to an industrial kibble diet using a Tunisian cohort of dogs. Analysis of the holobiont will shed light on the effect of diet intervention on the gut microbiota composition and the host response.

## Methods

### Animals

Thirty-two dogs were recruited from several farms belonging to Sidi Bouzid governorate in the center of Tunisia in the clinical practice of one of the co-authors (SR). The selected dogs were all semi-stray, which means that they were free within the perimeter of the farm, in contact with other species (cats, cattle, sheep, poultry, and horses) and self-nourished by hunting, what they found on the ground and sometimes fed by their owners (food waste, bread). The dogs were considered healthy based on normal clinical examination, normal blood cell count and biochemistry, and negative fecal flotation analysis for parasites.

The clinical investigator (SR) completed a questionnaire for each dog. The collected information included age, sex, reproductive status, breed, medical history, weight, and body condition score (BCS). A nine-point body condition scoring system was used, with BCS between 1 and 3 being “thin”, BCS between 4 and 5 being “ideal” and BCS over 6 being “overweight” or “obese” [13]. Dogs that had received medication in the last 6 months were not included. Dogs demographic data are summarized in Table 1. The detailed dietary habits of the participants were also collected to estimate the proportion of the main ingredients.

**Table 1:** Demographic data of the cohort

Groups	Breed	Sex (M/F)	Age (years)
			Median (1st quartile–3rd quartile)
Industrially-nourished (n = 22)	Mixed local breed (15)	13/9	3 (2–3.9)
	German shepherd (3)		
	Rottweiler (2)		
	Epagneul (1)		
	Pointer (1)		
Self-nourished (n = 10)	Mixed local breed (10)	8/3	3 (2–3.3)

### Dietary intervention

The 32 dogs were randomly divided into 2 groups. Group 1 (n = 10) served as a control and maintained the eating habits described above. Group 2 (n = 22) underwent a dietary transition to a kibble industrial food (Table 2).

**Table 2:** Composition and nutritional analysis of the kibble industrial diet

Ingredients	Corn, dehydrated poultry proteins, corn flour, animal fats, dehydrated beef and pork proteins, hydrolyzed animal proteins, corn gluten, wheat, beet pulp, rice, fish oil, soybean oil, mineral salts, yeasts and yeast components, crustacean hydrolysates (source of glucosamine) and cartilage hydrolyzate (source of chondroitin), additives
Nutritional analysis (dry matter)	Protein 25%, Fat content 17%, Crude ash 6.2%, Crude fiber 1.8%

**Fig. 1:**
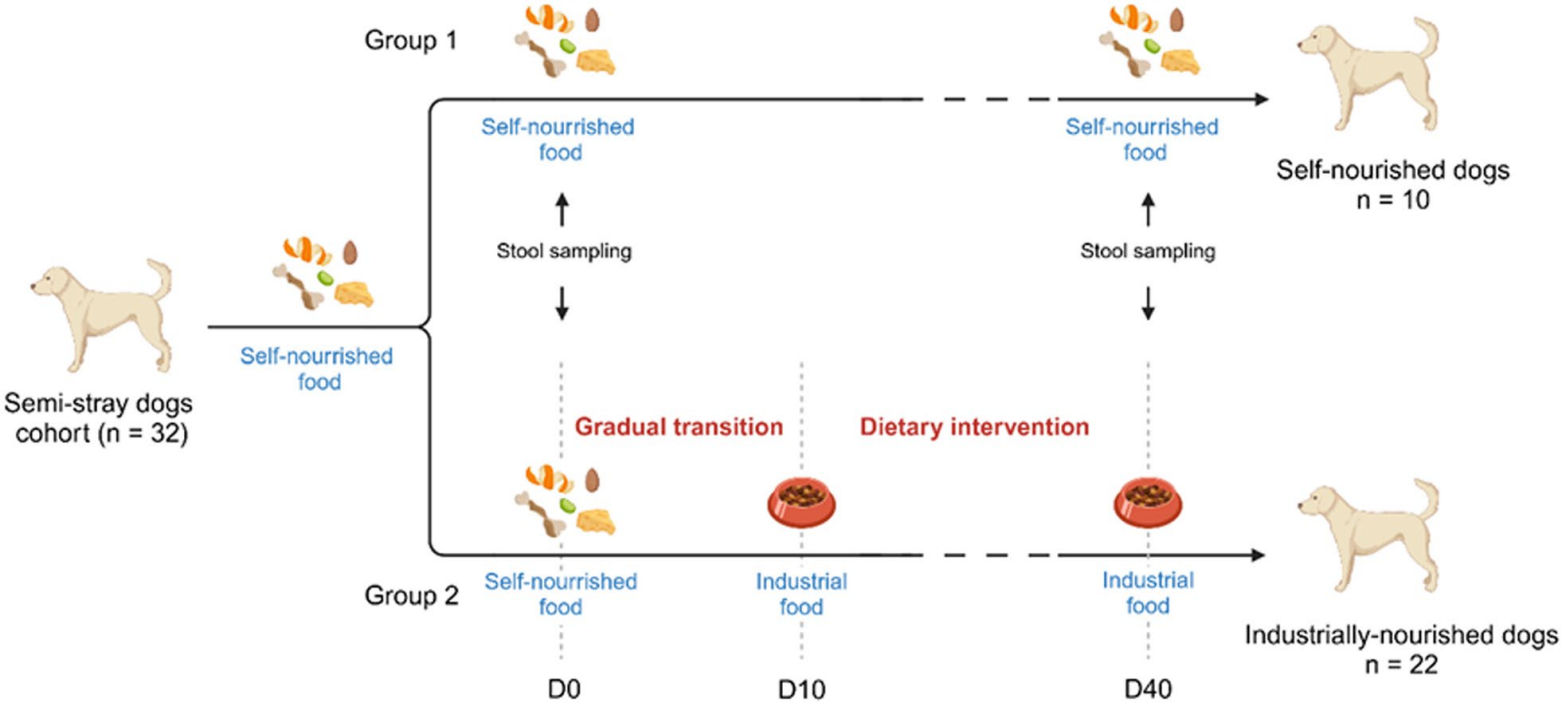
Study design

To ensure a gradual dietary transition in group 2, the supply of kibble was gradually increased over 10 days until full energy requirements were reached. Energy requirements (ER) were calculated according to the formula ER (kcal) = 156 × W ^0.67^ × 1.2 where W is the optimal weight. On the 10th day (D10), the dogs were confined in a large enclosure located inside the farm in order to cut off access to food sources other than the industrial food provided while maintaining physical activity and exposure to the usual environment (Fig. 1).

### Blood sample collection

Blood samples were collected in a Na-heparin tube on day (D) 0 and D40 from dogs fasted for 12 h to assess blood glucose and C-reactive protein concentrations. One drop of fresh blood from each sample was used for glycemia measurement. Subsequently, the blood was centrifuged at room temperature (825 × g for 10 min), and the plasma was carefully separated into Eppendorf tubes. The plasma samples were promptly frozen at -80 °C for subsequent analysis.

### Glycemia monitoring

Glycemia was assessed in dogs by measuring the concentration of glucose in the blood before and after diet change. One drop of blood from each sample was immediately used to measure glycemia using a portable digital glucometer (Sinocare Safe-Accu, Safecare Bio-tech, Yuhang, China) following the manufacturers' recommendations.

### C-reactive protein concentration

C-reactive protein (CRP) concentrations for each plasma sample were assessed using a commercially available method, as previously outlined by Carney et al. [14]. The measurement assays were conducted using the Canine C-Reactive Protein ELISA Kit from BD Biosciences, San Jose, CA, following the manufacturers' recommendations.

### Fecal sample collection and storage

Fecal samples were collected at D0 and D40 after spontaneous defecation, placed in a stool pot, stored in a cool box, and transferred within 3 h to the laboratory. Samples from each dog were stored at -80 °C until processing.

### DNA extraction and microbiota analysis

Metagenomic DNA was extracted from 200 mg of feces from each subject as described previously [15]. The amplification of the V3-V4 region was performed during 30 amplification cycles at 65˚C with designed primer pairs of the 16S ribosomal RNA locus (F343 5’CTTTCCCTACACGACGCTCTTCCGATCTACGGRAGGCAGCAG3’ and R784 5’GGAGTTCAGACGTGTGCTCTTCCGATCTTACCAGGGTATCTAATCCT3’). The obtained amplicons (510 bp) were purified and subjected to second PCR to add dual indices and sequencing adaptor requested for Illumina Miseq technology sequencing. The purification and loading of the amplification products onto the Illumina MiSeq cartridge were done according to the manufacturer’s instructions. Raw sequences were analysed using the bioinformatics pipeline FROGS (Find Rapidly OTU with Galaxy Solution) [16]. The sequences were clustered into operational taxonomic units (OTUs) of 97% similarity and a cut-off value of 0.03. OTUs were classified against the Silva Ribosomal Database at an 80% confidence interval using a naive Bayesian method. The OTU sequence data were calculated and presented as relative abundance ± SEM. α and β diversity analyses were accomplished with the phyloseq R package. We evaluated α-diversity using Observed, Shannon and Simpson indices. Community compositions profiles between groups were evaluated using permutational multivariate analysis of variances (PERMANOVA). Mann Whitney test was applied to analyze the differences in phyla, families, and genera between the two groups. Benjamini–Hochberg corrections were used to avoid false positives (significance threshold = 0.05) [17]. We calculated pairwise correlations between each specific genus and host parameters using Spearman’s nonparametric rank correlation coefficient. To calculate the Spearman correlations matrix, we used R 4.2.3, the corrplot and Hmisc package. We also performed statistical analysis of the microbiota data using the Rhea scripts pipeline [18].

### Fecal bile acids and short-chain fatty acids quantification

Fecal bile acids, including cholic acid (CA), chenodeoxycholic acid (CDCA), deoxycholic acid (DCA), lithocholic acid (LCA), hyodeoxycholic acid (HDCA), and ursodeoxycholic acid (UDCA), were quantified by liquid chromatography-tandem mass spectrometry (LC–MS/MS) from ~ 50 mg of fecal samples as described previously [19]..

Short-chain fatty acids (SCFA), including acetate, butyrate, isobutyrate, propionate, valerate and isovalerate, were quantified by gas chromatography–mass spectrometry from ~ 50 mg of fecal samples as described previously [20].

### Proteolytic activity assays

Fecal waters were prepared by adding 200 mg of thawed feces to 1 ml of assay buffer (20 mM Tris–HCl, 200 mM NaCl, pH 8). The samples underwent a 1-min sonication (10 s on / 10 s off, amplitude 39%), and were centrifuged at 4 °C, 10 000 rpm for 20 min. Fresh fecal water preparation were used for protease activity assays.

The proteolytic activity assessment consists of using specific chromogenic or fluorogenic substrates to monitor each of the specified activities, as detailed in Table 3. Azocasein was used as the substrate to quantify total activity, and the reaction was stopped with 10% (w/v) added trichloroacetic acid (TCA, Sigma). All reactions were performed in Tris–HCl buffer (20 mM Tris–HCl, 200 mM NaCl, pH 8). Fecal supernatants were diluted to the adequate concentration and 20 µl of the dilution were added to the reaction mixture containing 20 µl of the convenient substrate in a final volume of 200 µl. To confirm each protease family among the total proteolytic activity, inhibition assays were achieved using selective inhibitors of serine proteases, cysteine proteases and metalloproteases that are, respectively, phenylmethanesulfonyl fluoride (PMSF, 1 mM), E-64 (10 µM) and EDTA (1 mM). Other synthetic inhibitors were also used to determine Trypsin-like activity (100 µM), Chymotrypsin-like activity (0.1 µM), Neutrophil Elastase-like activity (NE, 10 µM), and Proteinase-3-like (PR3, 0.1 µM) activity. The reactions were then incubated in 96-well plates for 30 min at 37 °C. Absorbance (410 nm) or fluorescence (Excitation: 360 nm, Emission: 460 nm) were measured at room temperature on a Perkinelmer® plate reader.

**Table 3:** Specific substrates and inhibitors

Type	Specificity	Sequence
Substrates	Cysteine-like	AB2-Ile-Leu-Pro-Glu-ANB-NH2 (Chromogenic)
	Metalloprotease-like	MCA-Lys-Pro-Leu-Gly-Leu-DNP-Dpa-Ala-Arg-NH2 (Fluorogenic)
	Trypsin-like	AB2-Val-Val-Ser-Lys-ANB-NH2 (Chromogenic)
	Chymotrypsin-like	AB2-Lys-His-Trp-Tyr-ANB-NH2 (Chromogenic)
	NE-like	AB2-Met-Pro-Val-Ala-Trp-Glu-Tyr-(3-NO2)-NH2 (Fluorogenic)
	PR3-like	AB2-Tyr-Tyr-ABU-Asn-Glu-Pro-Tyr-(3-NO2)-NH2 (Fluorogenic)
	CatG-like	MCA-Phe-Val-Thr-Gnf-Ser-Trp-AB2-NH2 (Fluorogenic)
	Kallikrein	H-D-Pro-Phe-Arg-pNA · 2 HCl (Chromogenic)
	Chymase-like	N-succinyl-AAPF-pNA (Chromogenic)
Inhibitors	Cysteine-like	E-64
	Metalloproteases	EDTA
	Serine-like	PMSF
	Trypsin-like	Soybean Trypsin Inhibitor (STI)
	Chymotrypsin-like	Nα-Tosyl-Phe Chloromethyl Ketone (TPCK)
	NE-like	N-(Methoxysuccinyl)-Ala-Ala-Pro-Val-chloromethyl ketone
	PR3-like	Bt-Val-Tyr-Asp-nValP(O-C6H4-4-Cl)2
	CatG-like	Ac-Phe-Val-Thr-PhgP(4-guanidine)-(OC6H4-4-S-Me)2

### Statistical analyses

Shapiro–Wilk testing was used to assess the normality of quantitative variables. Normally distributed variables are presented as mean and standard deviation (SD), while non-normally distributed results are presented as median and interquartile range. The R 4.2.3 software was used for statistical analysis. Differences in the study parameters between dogs before and after diet change and between groups were determined using the Mann Whitney test. Statistical significance is indicated as * for *p* < 0.05, ** for *p* < 0.01, and *** for *p* < 0.001. Statistical significance was considered at *p* < 0.05.

## Results

### Cohort description

The characteristics of the two groups in terms of body weight, and BCS, at D0 and D40, are reported in Table 4. The recruited dogs display similar characteristics in the 2 groups at D0. They are mostly local breed dogs and young adults with a median age of 3 years. The gender distribution shows a statistically insignificant difference between the groups (males represent 59% of the industrially-nourished group and 72% of the self-nourished group). The animal included were medium-sized (median weight of 16.5 kg and 17 kg in the industrially-nourished and self-nourished groups, respectively). The median BCS at D0 was 3, revealing that animals were thin at the beginning of the study.

**Table 4:** Comparison of body weight and body condition score at D0 and D40

Groups	Body weight (kg)		Body condition score*	
	Median (1st quartile–3rd quartile)		Median (1st quartile–3rd quartile)	
	D0	D40	D0	D40
Industrially-nourished (n = 22)	16.5^a^ (13.3–20.8)	20.8^a^ (17–25.8)	3^a^ (3–4)	4.5^a^ (4–5)
Self-nourished (n = 10)	17^b^ (14.8–19.8)	18^b^ (16–20)	4 (3.5–4)	4 (4–4)

Significant differences (*p* < 0.05) between groups are shown with the same superscript symbols (a–b)

*Body condition score was established based on a maximal mark of 9. Ideal body condition score is between 4 and 5

Moreover, the dietary questionnaire completed by the owners allowed us to estimate the typical diet of semi-stray dogs. Table scraps are given daily and are typically those from Mediterranean cuisine composed of wheat (bread, pasta, or semolina), olives and olive oil, bones of mutton, beef, and poultry, fruits and vegetables (peeling of carrots, onions, garlic, …) (Table 5).

**Table 5:** Description of the food ration of semi-stray dogs

Food category	Main ingredients	Ingestion frequency
Table scraps	Whole grains, bread, beans, nuts and seeds	Daily
	Peeling of vegetable and fruits	Daily
	Olive Oil	Daily
	Dairy products mainly in the form of fermented products (yogurt, cheese …)	Once a week
	Bones	Once a week
Hunting	Rabbits, wild pigs	Once a week
Carrion	Carrion, placenta	Once a month

As reported in Table 4, body weight significantly increased between D0 and D40 in both groups (+ 26% body weight in the industrially-nourished group and + 6% body weight in the self-nourished group). The industrially-nourished group also presents a significant increase in BCS at D40 compared to D0, ranging from a median of 3 to 4.5, which ranged from “thin” to “ideal”.

### Effect of diet change on blood glucose and CRP concentrations

In our study, we characterized the blood concentration of glucose and CRP to evaluate the impact of the selected diet on glucostasis and the inflammatory response. We did not find significant differences in glycemia before and after diet change nor with the control group. Similarly, CRP levels were not significantly altered by diet change (Supplementary Fig. 1).

### Characterization of the fecal SCFA profile in semi-stray dogs before and after diet change

**Fig. 2:**
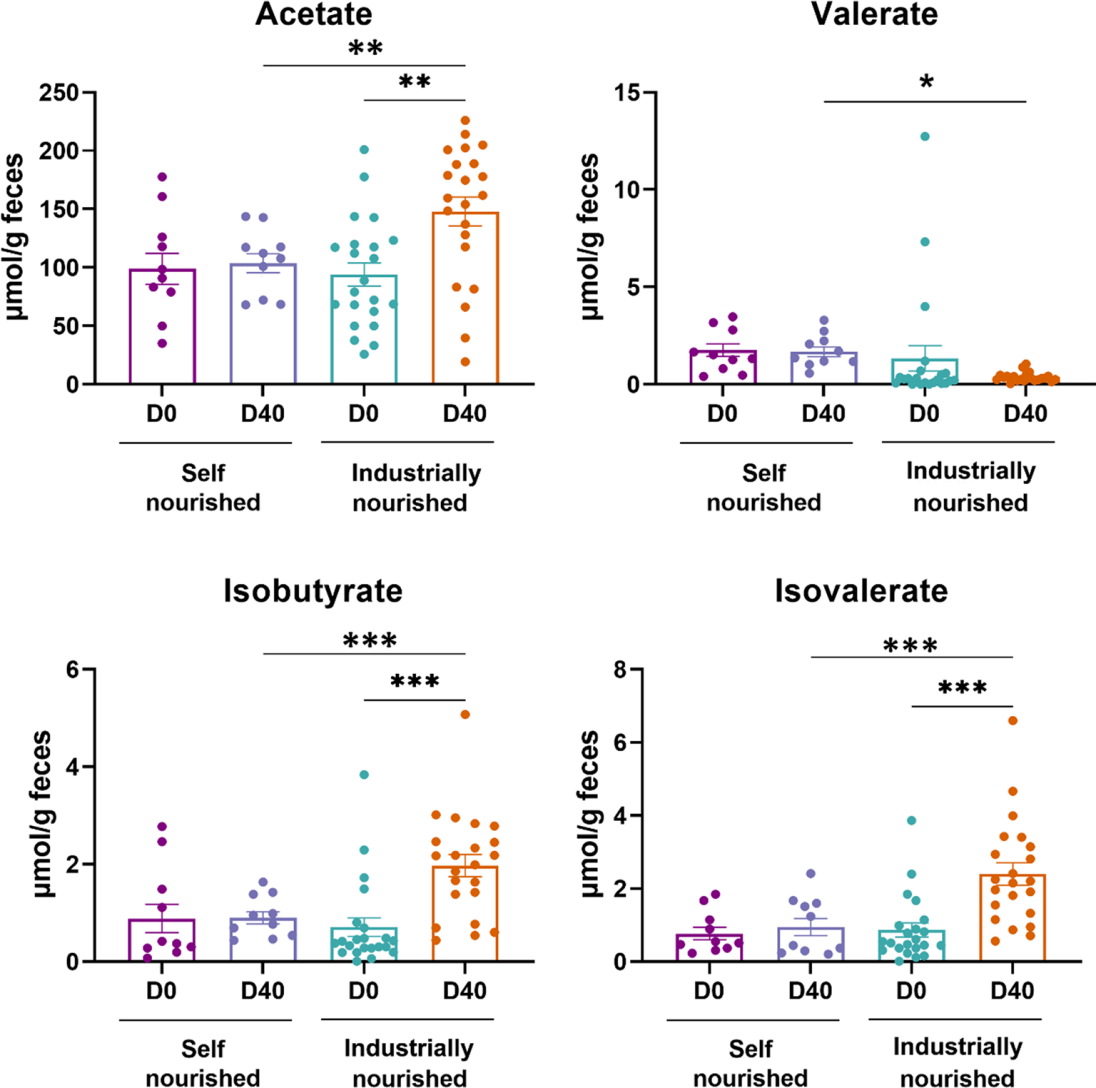
Characterization of fecal SCFA profile in self-nourished and industrially-nourished dogs before (D0) and after (D40) diet change. Data are presented as means ± standard error of the mean (SEM). Statistical analyses were performed using Kruskal–Wallis followed by Dunn’s test to compare SCFA profile in semi-stray dogs before and after self-nourished and industrially-nourished diet change. *p < 0.05; **p < 0.01; ***p < 0.001. SCFA, short-chain fatty acids

Our data showed that the fecal concentration of isobutyrate, acetate and isovalerate increased by 2.8-fold (*p* < 0.001), 1.6-fold (*p* < 0.01) and 2.8-fold (*p* < 0.001) respectively in semi-stray dogs after the diet change (Fig. 2). No statistical difference was observed between valerate, butyrate and propionate concentration before and after the dietary intervention (Supplementary Fig. 2).

### Characterization of fecal bile acids profile in semi-stray dogs before and after diet change

**Fig. 3:**
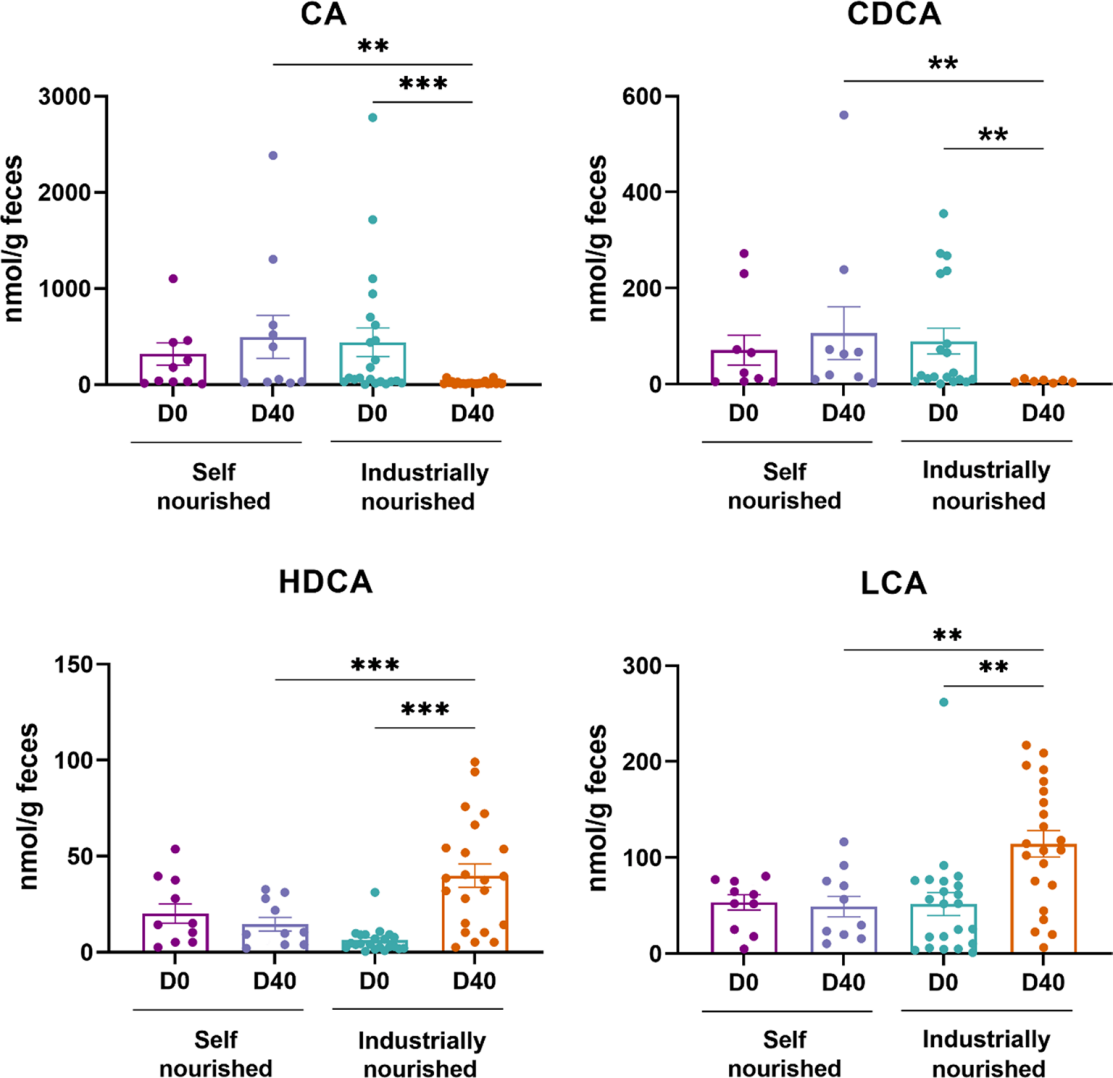
Quantification of fecal BAs level in self-nourished and industrially-nourished dogs before (D0) and after (D40) diet change. Data are presented as means ± SEM. Statistical analyses were performed using Kruskal–Wallis followed by Dunn’s test to compare bile acid profile in semi-stray dogs before and after self-nourished and industrially-nourished diet change. **p < 0.01; ***p < 0.001. BAs, bile acids

The analysis of BA profiles revealed a significant decrease of primary BA levels, namely cholic acid (CA) and chenodeoxycholic acid (CDCA) in dogs fecal samples after diet change by 22.5-fold (*p* < 0.001) and 14-fold (*p* < 0.01) respectively (Fig. 3). Moreover, our results also show an increase in two secondary BA namely hyodeoxycholic acid (HDCA; 4.4-fold higher) and lithocholic acid (LCA; 2.2-fold higher) in dog fecal samples at D40 compared to D0 (Fig. 3). On the contrary, fecal levels of two other secondary BA deoxycholic acid (DCA) and ursodeoxycholic acid (UDCA) were unchanged at D40 (Supplementary Fig. 3).

### Fecal proteolytic profile in semi-stray dogs before and after diet change

**Fig. 4:**
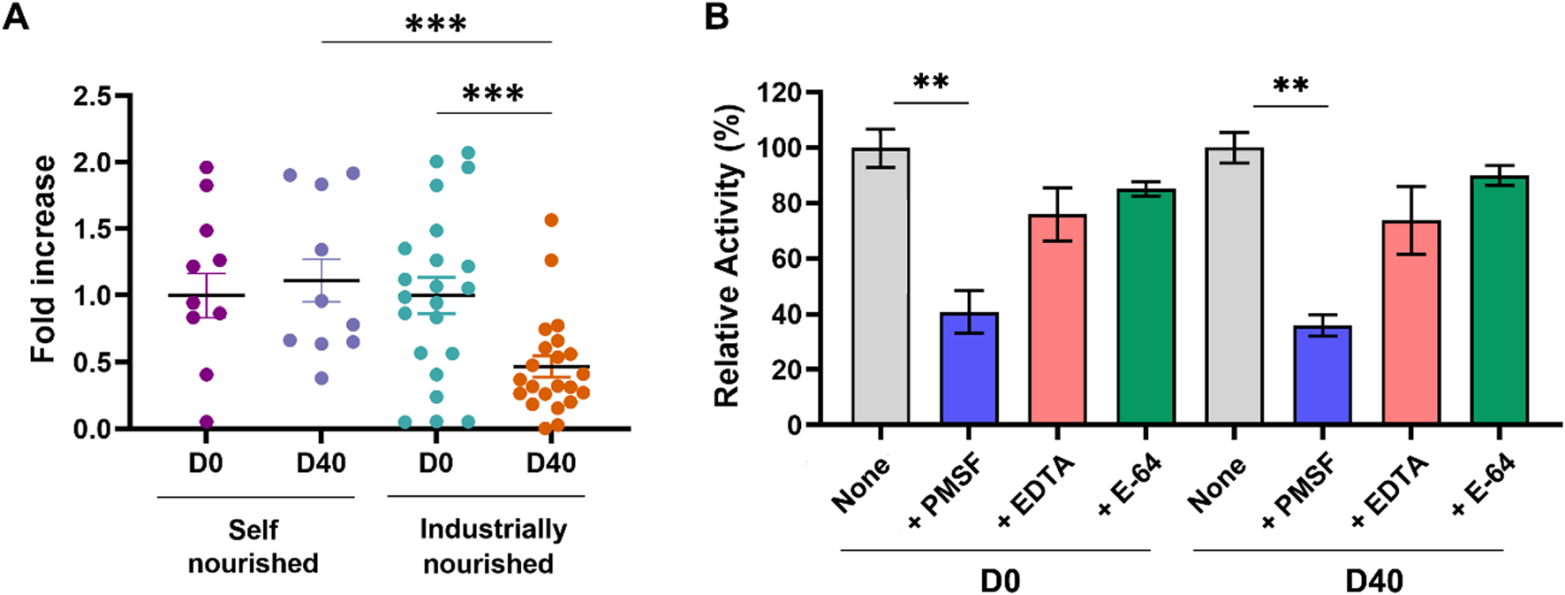
Fecal proteolytic activity in semi-stray dogs before (D0) and after (D40) self-nourished and industrially-nourished diet change. (**A**) Total fecal proteolytic activity in semi-stray dogs before and after self-nourished and industrial diet. (**B**) Relative proteolytic activity in the presence and absence of inhibitors (PMSF, EDTA, and E-64) for protease families. "Fold increase" represents the factor by which the activity has increased compared to the reference (average proteolytic activity measured in semi-stray dogs before diet change). Relative activity corresponds to the defined maximum activity, set as 100%. Data are presented as means ± SEM. Kruskal–Wallis followed by Dunn’s test was used to compare different proteolytic activities in semi-stray dogs before and after self-nourished and industrially-nourished diet change. The Mann–Whitney test was used to compare total activity in the presence and absence of the used inhibitors. **p < 0.01, ***p < 0.001

Analysis of total proteolytic activity revealed a two-fold decrease after the diet change compared to samples obtained before the diet change (*p* < 0.001; Fig. 4A). Several specific protease inhibitors were tested to determine the respective contribution of protease families to proteolytic activities (Fig. 4B). The proteolytic activity was significantly reduced by approximately 60% in fecal samples of both groups (*p* < 0.01) in the presence of PMSF, a broad-spectrum SP inhibitor, demonstrating a major contribution of SPs. In the presence of the metalloprotease inhibitor EDTA, proteolytic activity in Group 2 was slightly reduced by 20% (not statistically significant). When using the cysteine protease inhibitor E-64, we observed only a 16% decrease in the activity (not statistically significant).

**Fig. 5:**
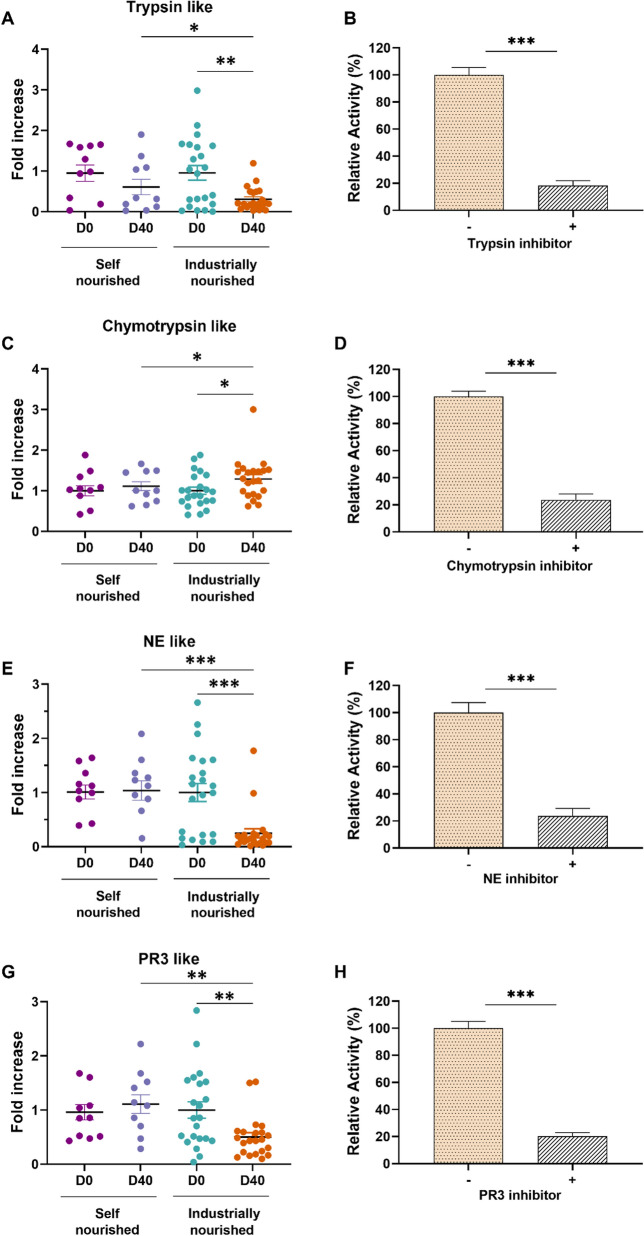
Characterization of fecal proteolytic activity profile in self-nourished and industrially-nourished dogs before and after diet change. (**A**) Trypsin-like activity. (**B**) Trypsin-like activity in the presence and absence of their specific inhibitor. (**C**) Chymotrypsin-like activity. (**D**) Chymotrypsin-like activity in the presence and absence of their specific inhibitor. (**E**) Neutrophil elastase-like activity (NE-like). (**F**) Neutrophil elastase-like activity in the absence and presence of their specific inhibitor. (**G**) Proteinase 3-like activity (PR3-like). (**H**) Proteinase 3-like activity in the absence and presence of their specific inhibitor. "Fold increase" represents the factor by which the activity has increased compared to the reference (average proteolytic activity measured in free-roaming dogs). Relative activity corresponds to the defined maximum activity, set as 100% (without (-) inhibitor). Data are presented as means ± SEM. Kruskal–Wallis followed by Dunn’s test was used to compare different proteolytic activities in semi-stray dogs before and after self-nourished and industrially-nourished diet change. The Mann Whitney test was used to compare proteolytic activities in the presence and absence of the used inhibitors. *p < 0.05; **p < 0.01; ***p < 0.001

To further characterize the protease contributing to the SP activity in semi-stray dogs, we used specific substrates for subfamilies of SPs. We were able to demonstrate a significant decrease of 70% of trypsin-like activity in dogs after diet change when compared to D0 (*p* < 0.01; Fig. 5A) and the control group (*p* < 0.05; Fig. 5A). We used specific inhibitors for each targeted protease subfamily to confirm the specificity of the designed substrates. Our results showed a significant 88% decrease in proteolytic activity in the presence of trypsin inhibitor (*p* < 0.001; Fig. 5B). Using specific substrates for chymotrypsin, we showed a significant increase of chymotrypsin activity by 1.3-fold higher in semi-stray dogs after a diet change when compared to D0 and control group (*p* < 0.05; Fig. 5C). This result was confirmed by a decreased of 76% in chymotrypsin activity in the presence of its specific inhibitor. Similarly, we also evaluated elastolytic activity (neutrophil elastase-NE and proteinase 3-PR3) in the different samples. Interestingly, we showed a decrease of 88% and 50% in fecal NE-like and PR3-like activity after diet change when compared to D0 (*p* < 0.001; Fig. 5E for NE-like and *p* < 0.01; Fig. 5G for PR3). Furthermore, we validated the increased activity using specific inhibitors for NE and PR3. As shown in Fig. 5F, H, we noted a decrease in proteolytic activity of 76% and 79% for NE-like and PR3-like activities respectively in the presence of their specific inhibitors (*p* < 0.001; Fig. 5F, H).

On the other hand, the use of specific substrates for other serine proteases (cathepsin-G, kallikrein, chymase-like), metalloproteases, and cysteine proteases showed no significant difference in proteolytic activity between dogs before, after diet change and control group (data not shown).

### Effect of diet change on fecal microbiota composition in semi-stray dogs

**Fig. 6:**
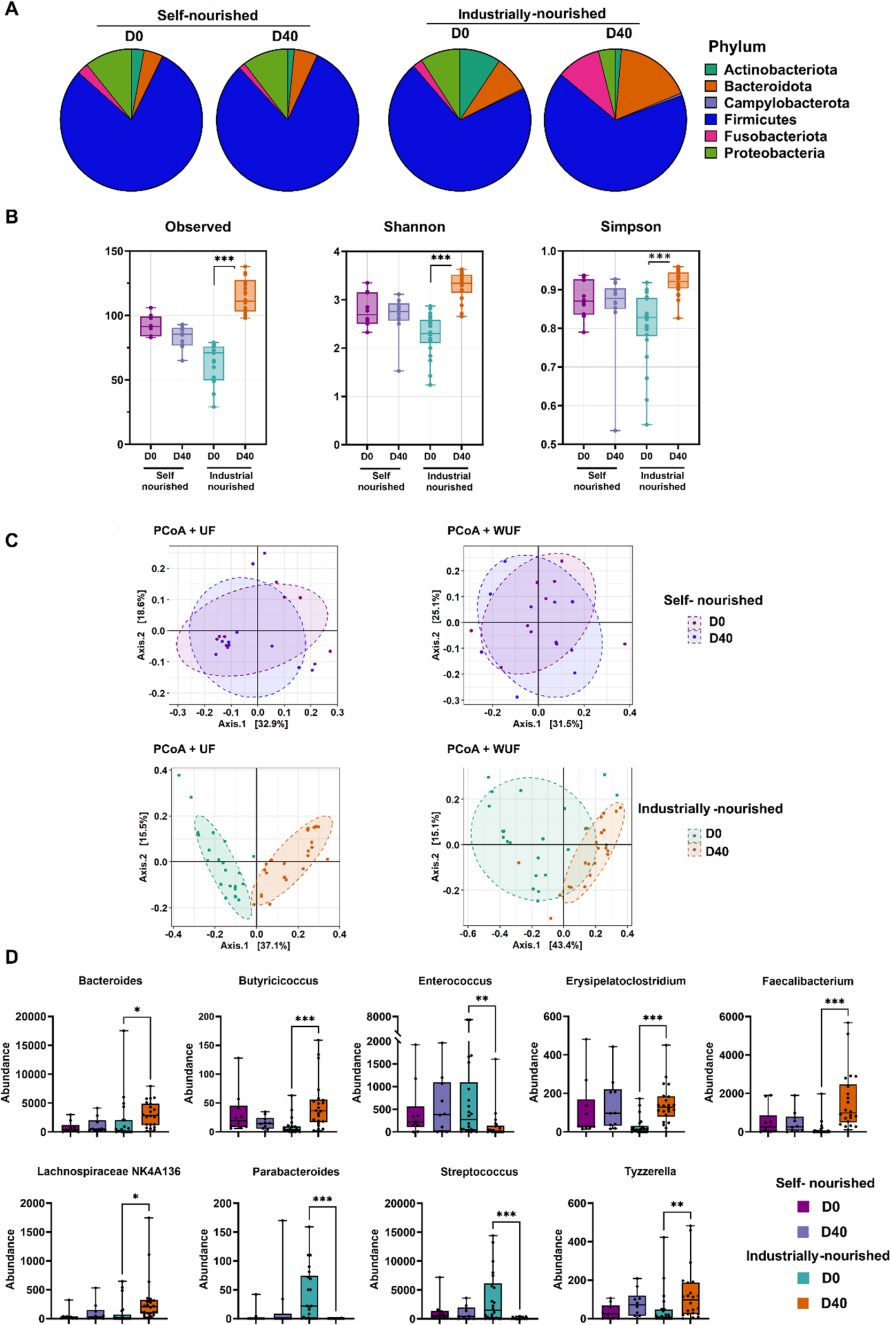
Gut microbiota analysis. (**A**) fecal microbiota composition at phylum level. (**B**) Alpha diversity indices, which take into account the community richness, evenness and diversity, was measured by three different indexes: Observed number of OTUs, Shannon and Simpson. (**C**) Principle Coordinate Analysis (PCoA) with unweighted unifrac (UF) distance matrices and weighted Unifrac (WU) matrix for semi-stray dogs before and after self-nourished and industrially-nourished diet change respectively. (**D**) Genera differentially abundant in semi-stray dogs before (D0) and after (D40) industrially-nourished diet change. Data are presented as means ± SEM. Statistical analyses were performed with the Mann–Whitney. Statistical significance is denoted as follows: *for *p* < 0.05, **for *p* < 0.01, and ***for *p* < 0.001

A significant modification of the gut microbiota composition in industrially-nourished semi-stray dogs at D40 compared to D0 was observed. Analysis of gut microbiota composition at the phylum level demonstrates an increase in the abundance of *Bacteroidota* and *Fusobacteriota* along with a decrease in the abundance of *Actinobacteria* and *Proteobacteria* phyla (Fig. 6A). Our data shows a significant increase in Shannon and Simpson indexes (*p* < 0.001) in group 2 dogs at D40 compared to D0, demonstrating an increase in bacterial abundance and richness (Fig. 6B). Beta diversity analysis, based on the Principal Coordinate Analysis (PCoA), revealed a distinct clustering of the gut microbiota between dogs fecal samples before and after the transition from the shelf-nourished diet to an industrial diet (PERMANOVA; *p* = 0.0004) (Fig. 6C).

As shown in Fig. 6D, we demonstrated a shift in microbiota composition at the genus level with the enrichment of *Bacteroides* (*p* < 0.05) and particularly of beneficial bacteria such as, *Butyricicoccus* (*p* < 0.001), *Erysipelatoclostridium* (*p* < 0.001), *Lachnospiraceae* NK4A136 (*p* < 0.05), *Tyzzerella* (*p* < 0.01) and *Faecalibacterium* (*p* < 0.01). In contrast, we also showed a decrease in bacterial abundance of *Parabacteroides* (*p* < 0.001), *Enterococcus* (*p* < 0.01), and *Streptococcus* (*p* < 0.001) genera.

### Gut microbiota association with differentially upregulated biological functions

**Fig. 7:**
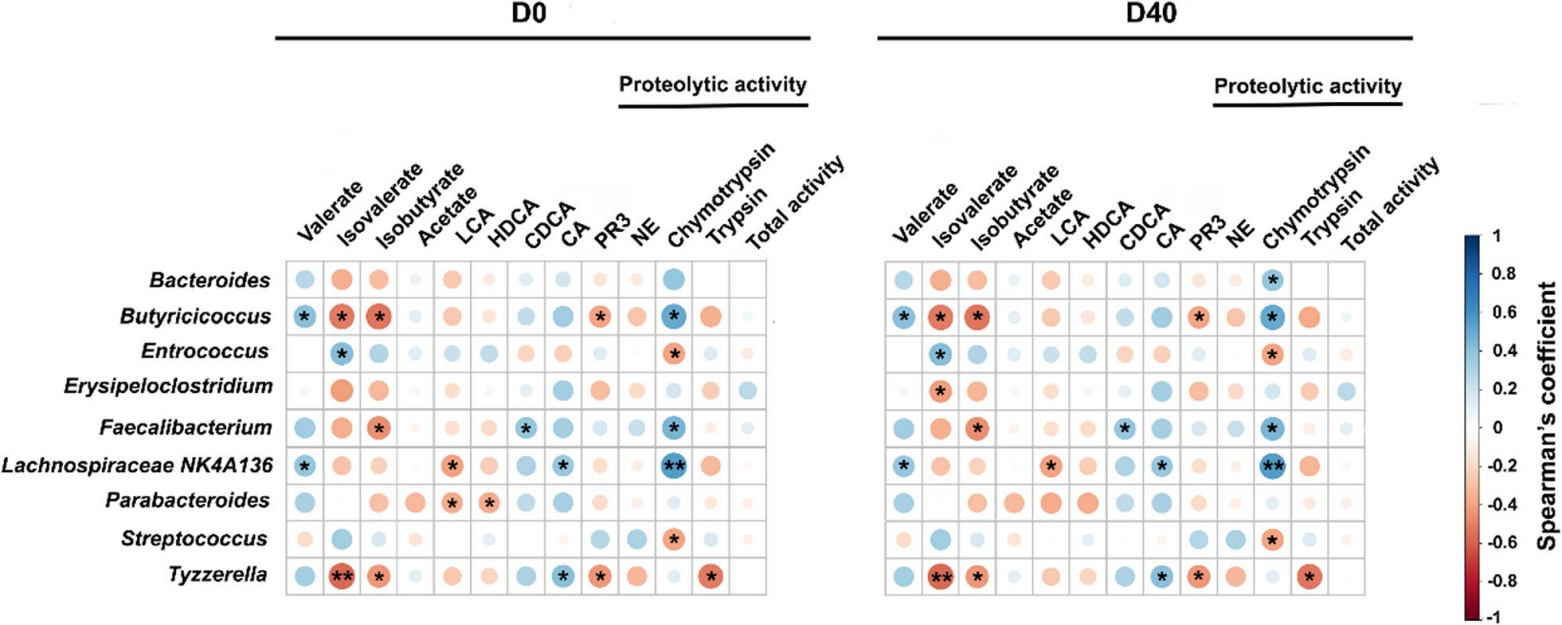
Spearman correlations between microbial populations and SCFAs, bile acids and proteolytic activities. For correlation analysis, the colors represent the correlation's nature, where dark blue signifies a robust positive correlation, and dark red signifies a substantial negative correlation. Statistical analyses were performed with Mann–Whitney. Statistical significance is denoted as follows: *p < 0.05, **p < 0.01, and ***p < 0.001, all after false discovery rate correction

We further investigated the relationship between altered gut microbiota and biological functions by performing a correlation analysis (Fig. 7). For SCFA, our data showed a significant negative correlation between isobutyrate and *Butyricicoccus, Faecalibacterium* and *Tyzzerella* genera abundance. Moreover, we observed a significant negative correlation for isovalerate and *Butyricicoccus* and *Tyzzerella* genera. Only after diet change, we observed a significant negative correlation between isovalerate and *Erysipeloclostridium*.

Regarding BAs, we noticed a significant negative correlation between *Parabacteroides* abundance and two secondary BAs levels, namely LCA and HDCA. This negative correlation becomes non-significant at D40 within the industrially-nourished group.

Our data showed a significant positive correlation between chymotrypsin activity and 3 bacterial genera: *Butyricicoccus, Faecalibacterium,* and *Lachnospiraceae* NK4A136 (Fig. 7). Interestingly, the positive correlation found between *Bacteroides* abundance and chymotrypsin activity reached statistical significance in dogs after diet intervention. Besides, we also observed a significant negative correlation between *Tyzzerella* genus and PR3 and trypsin activities.

## Discussion

The influence of diet on the composition and function of the gut microbiota is well-known in humans and animals [21, 22]. However, most studies are cross-sectional and carried out in industrialised countries in Europe [23] or North America [24] or on animals in laboratory facilities. We studied the impact of diet in a cohort of dogs living in their natural environment in North Africa by carrying out a controlled dietary trial and measuring the impact in terms of composition and targeted functions of their intestinal microbiota.

The eating habits of the animals in our cohort are typical of MD, which could make the Tunisian semi-stray dog a good model for studying the role of this diet on health (Table 5). The industrial food used in our study is a food specially formulated to meet the nutritional recommendations from the European Pet Food Industry Scientific Advisory Board. This food, therefore, contrasts with natural food because it is complete and balanced. It is not comparable with WD because it does not contain refined sugars, fats and animal proteins in excessive quantities. The proposed industrial food also contains the recommended amount of fiber. For animals included in the industrially-fed group, we chose to make a gradual dietary transition over 10 days because an abrupt transition can lead to diarrhea and significant disruption of the intestinal microbiota [25]. The size of the control group was reduced during the experiment (from 22 to 10 dogs) due to constraints linked to the fact that the study was carried out in real farm conditions. Therefore, we excluded in the control group the animals for which we had doubts about whether the owners were following the instructions carefully. While groups are not even which can constitute a limitation, the statistical power of our study remains significant.

During the study period, dogs from both groups showed significant weight gain and, for the industrially-fed group, a significant increase in BCS (Table 4). The owners of the self-fed dogs may have felt pressure linked to veterinary supervision, leading to a more abundant food during the observation period. In addition, the energy intake of the industrially-nourished group was calculated based on the optimal weight while the animals were “thin”. Moreover, glycemia is not affected during industrial diet implementation, suggesting that it provides medium energy levels that sustain glucostasis (Supplementary Fig. 3A). Similarly, inflammation response as assessed by blood CRP levels was not altered in dogs during dietary intervention (Supplementary Fig. 3B).

Short-chain fatty acids play a crucial role in maintaining overall health, particularly in the context of gut health and metabolic processes. SCFA are an energy source for colonocytes, improve gut barrier function and lipids and carbohydrates metabolisms, and reduce luminal pH, providing a resistance mechanism against pathogens [26–28]. It is important to note that while SCFAs offer numerous health benefits, their effects are complex and can vary depending on factors such as diet, gut microbiota composition, and individual health status [29]. Herein, we evaluated their production before and after diet change. The measurement of fecal SCFA revealed a significant increase in the contents of acetate, isobutyrate and isovalerate after the change to industrial food compared to D0 and the control group. This effect may be due to the evolution of the populations of SCFA-producing bacteria, the abundance of the fermentation substrate constituted by the non-digestible fiber and starch contained in the industrial kibbles, or a combination of both mechanisms. In fact, the abundance of known bacterial SCFA-producers increases upon diet change (e.g., *Bacteroides*, *Feacalibacterium,* *Butyricicoccus*). These findings align with the work of Jha and Berrocoso [30] in swine and Myint et al. [31] in dogs. Acetate is particularly important as it has beneficial effects on host energy and substrate metabolism, affecting appetite and insulin sensitivity [32]. Acetate supplementation decreases weight gain induced by a high-fat diet in mice [33]. Several in vivo studies reported that acetate can modulate satiety via the secretion of glucagon-like peptide-1 and peptide YY, two gut-hormones involved in satiety and appetite regulation [34, 35]. In addition, acetate can modulate insulin response and, hence, glucose homeostasis [36]. Therefore, an industrial diet's beneficial effects may be partly mediated by the increased acetate concentration. In contrast to other SCFAs, we did not find a significant difference in butyrate or propionate after the diet change. This was an unanticipated finding, as the increased bacterial groups in industrially-nourished dogs are considered to be butyrate producers.

The measurements of fecal BAs showed a significant decrease in primary BAs (CA and CDCA) and an increase in specific secondary BAs (HDCA and LCA) in the group fed with industrial food at D40 compared to D0 and compared to the control group. Our data suggest an increase in the abundance of bacteria able to convert primary BA into secondary BA after dietary change, particularly the conversion of CDCA to LCA. This is supported by the observed increase in the abundance of *Erysipelatoclostridium* and *Lachnospiraceae* NK4A136, two genera known to perform 7α-dehydroxylation [37]. After diet change, HDCA levels are increased indicating a favoured conversion of LCA to HDCA in dogs. Interestingly, several studies have reported beneficial effect of HDCA including anti-inflammatory properties [38, 39]. Our data suggest that *Faecalibacterium*, *Lachnospiraceae,* and *Bacteroides*, known for their bile salt acid activity, as candidate bacterial genera that could contribute to the observed shift in bile acid profile contributing to the metabolic benefit provided by the dietary intervention [40, 41].

In the present study, fecal proteolytic activity was decreased in the group fed with the industrial food compared to before dietary intervention and the control group. Analysis of the profile showed that the fecal proteolytic activity was mainly due to the SP activity and that the trypsin-like, NE-like and PR3-like activities are significantly reduced at D40. A reduction in fecal proteolytic activities may be a beneficial effect sought because proteolytic hyperactivity has been demonstrated to be involved in the pathogenesis of IBD in humans and dogs [42, 43]. Diet could therefore constitute an interesting tool for regulating proteolytic activity. Furthermore, the analysis of correlations with the composition of the microbiota (Fig. 7) showed a positive correlation with some bacterial genera (*Faecalibacterium*, *Lachnospiraceae*) and a negative one with others (*Butyricioccocus* for PR3, *Enterococcus*, *Streptococcus* and *Tyzzerella*). In humans with irritable bowel syndrome, Edwinson et al. documented a correlation between SPs activity and gut microbiota composition [10]. The regulation of fecal proteolytic activity may partly rely on the modulation of the microbiota towards bacterial genera expressing regulatory serpins [44].

The change from natural to industrial food led to significant changes in the intestinal microbiota composition as shown by an increase in alpha diversity and PCoA analysis (Fig. 6C). As shown in Fig. 6D, we noted an enrichment of beneficial bacteria such as *Bacteroides*, *Butyricicoccus*, *Erysipelatoclostridium*, and *Faecalibacterium*. These genera are known for their pivotal roles in maintaining a balanced and thriving gut microbiome. *Bacteroides* are important for breaking down complex carbohydrates and aiding in nutrient absorption, contributing to overall digestive efficiency [2]. *Butyricicoccus* [45] are producers of short-chain fatty acids such as butyrate, which are not only energy sources for colonocytes but also have anti-inflammatory properties and support intestinal barrier function. *Faecalibacterium* [2, 4] is a well-recognized butyrate producer and is linked to anti-inflammatory effects in the gut. The increase in these beneficial genera reflects a positive shift in the gut microbiota composition, potentially leading to improved digestive health, enhanced immune function, and a reduced risk of gastrointestinal disorders. In contrast, we also observed a decrease in bacterial abundance of *Enterococcus* (*p* < 0.01), and *Streptococcus* (*p* < 0.001) genera. These genera are commonly associated with imbalances in the gut microbiome [5–7]. Our finding are consistent with a previous study comparing dogs fed with bones and raw food (BARF) (uncooked meat and bones) to dogs fed with a commercial diet [29]. In fact, dogs fed a commercial diet shows higher abundance of *Faecalibacterium, Erysipelotrichaceae* and *Lachnospiraceae* and lower *Streptococcus* and *Enterococcus* abundance compared to BARF-fed dogs. In this context, dogs fed with BARF diet was characterized by a higher richness and diversity in their gut microbiota compared to the commercial diet [46]. The shotgun metagenomic analysis carried out by Alessandri et al. on wolves and domestic dogs showed that the microbiome evolved during domestication to adapt to a dietary intake rich in carbohydrates. These results indicate that the domestication of dogs significantly shaped intestinal microbial populations in favor of fibrolytic populations [47].

The transition from a natural diet in Tunisian semi-stray dogs to an industrial kibble diet was accompanied by beneficial changes in the composition of the microbiota and in the evaluated functional aspects (increased SCFA synthesis and BAs bioconversion, and reduction in fecal proteolytic activities). All these effects are considered beneficial because they help maintain intestinal and immune homeostasis [48, 49]. The results of this study reinforce the importance of diet beyond all other environmental factors. Indeed, the study was carried out in a complex environment located in a developing country with many other animal species (cats, cattle, sheep, poultry, and horses) in contact with dogs. The design of our study made it possible to maintain the impact of inter-species microbial flows throughout the experiment, and thus showed that diet has a very high impact. Surprisingly and contrary to what one may believe, the transition from a natural food found in the dog's environment to a highly processed industrial food was accompanied by beneficial changes in the host's health. Likely, it is not so much whether it is processed or not that has an impact, as much as the nutritional balance of the food. Indeed, industrial foods meet the nutritional recommendations formulated at the international level. The comparison of the macronutritional analysis between the self-nourished group and the industrially-nourished group is difficult because the composition of the semi-stray dogs' intake is estimated. However, industrial food is likely richer in indigestible fiber and starch, thus making it possible to promote a diverse and functionally beneficial microbiota.

## Conclusions

In conclusion, this study demonstrates increased fecal SCFA and secondary BAs concentration, decreased proteolytic activity, and significant alteration of gut microbiota composition in Tunisian semi-stray dogs upon dietary intervention. These findings show the beneficial effect of changing from a natural diet to a nutritionally balanced industrial food on microbiota composition, functions and dog health. Further studies are needed to determine whether dietary intervention by modulating the composition and function of the gut microbiota can be used as a powerful strategy to restore altered microbial functions leading to a new strategy to prevent some diseases (*e.g.*, digestive inflammation) or improve clinical signs.

## Supplementary Information

Additional file 1.

## Data Availability

The data that support the findings of this study are openly available in Biostudies database at https://www.ebi.ac.uk/biostudies/, reference number S-BSST1356 (16S rRNA sequencing dataset).

## Abbreviations

BAs: Bile acids
BCS: Body condition score
BARF: Bones and raw food
CDCA: Chenodeoxycholic acid
CA: Cholic acid
CRP: C-reactive protein
DCA: Deoxycholic acid
ER: Energy requirements
HDCA: Hyodeoxycholic acid
IBD: Inflammatory bowel disease
LCA: Lithocholic acid
MD: Mediterranean diet
NE: Neutrophil elastase
OTUs: Operational taxonomic units
PERMANOVA: Permutational multivariate analysis of variances
PMSF: Phenylmethanesulfonyl fluoride
PCoA: Principal coordinate analysis
PR3: Proteinase-3
SP: Serine protease
SCFA: Short-chain fatty acids
SD: Standard deviation
SEM: Standard error of the mean
UF: Unweighted unifrac
UDCA: Ursodeoxycholic acid
WU: Weighted unifrac
WD: Western diet
