# Supplementary material for: Industrial diet intervention modulates the interplay between gut microbiota and host in semi-stray dogs
Source: Anim Microbiome. 2024 Nov 21;6:69. doi: 10.1186/s42523-024-00357-w (PMC11580502; doi:10.1186/s42523-024-00357-w)
Supplement: Supplementary file 1 — Additional file 1. [file 42523_2024_357_MOESM1_ESM.docx]

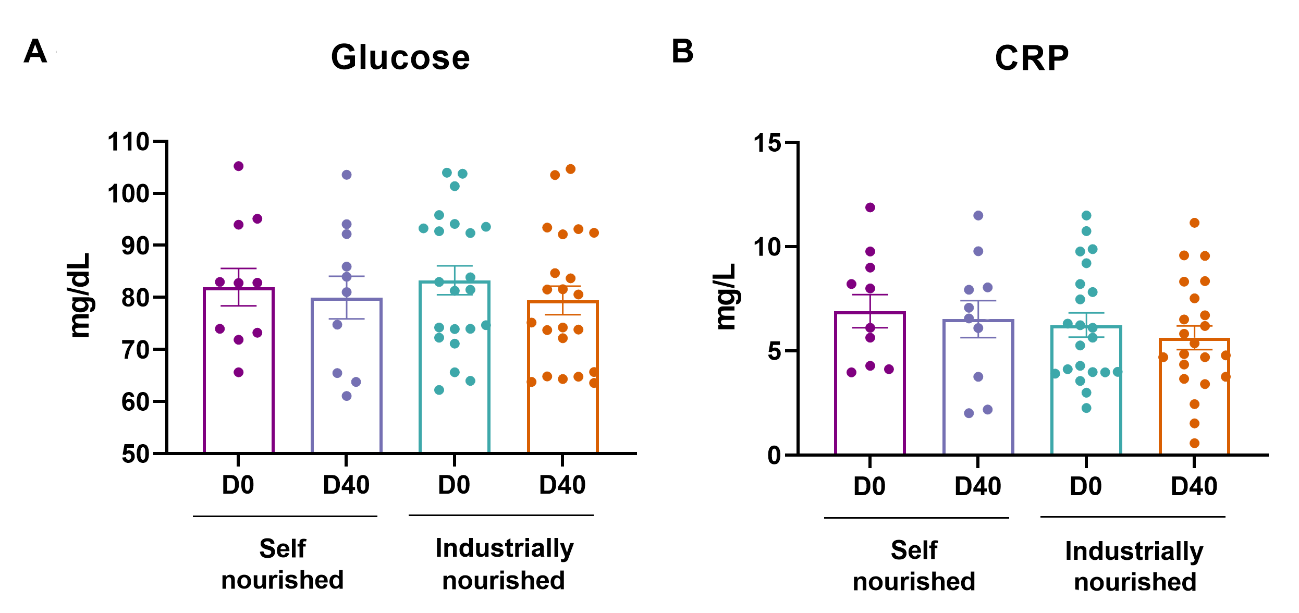


**Supplementary Figure 1** Blood glucose and CRP levels in semi-stray dogs before and after self-nourished and industrial diet change. (**A**) Glycemia level in in semi-stray dogs before and after self-nourished and industrial diet change. (**B**) CRP concentration in semi-stray dogs before and after self-nourished and industrial diet change. Data are presented as means ± SEM. CRP, C-reactive protein.


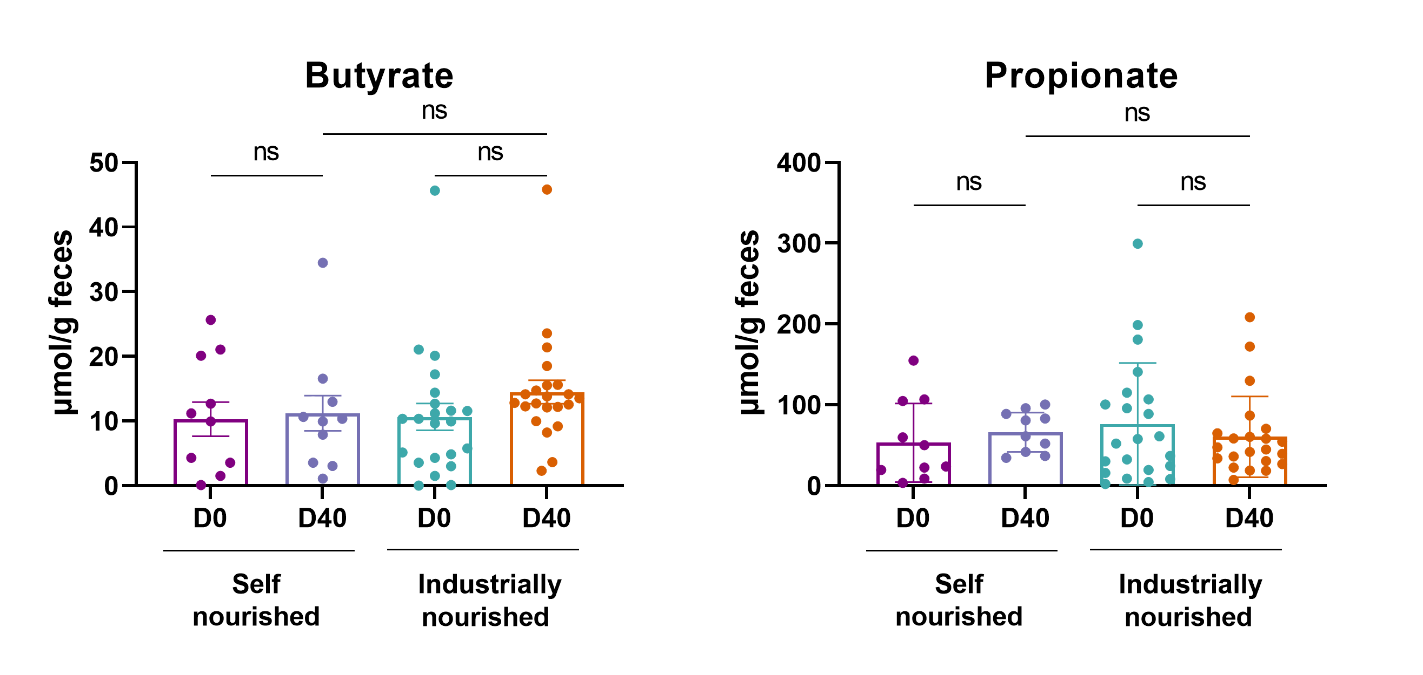


**Supplementary Figure 2** Characterization of fecal SCFA profile in semi-stray dogs before and after self-nourished and industrial-nourished diet change. Data are presented as means ± SEM. Statistical analyses were performed using Kruskal-Wallis followed by Dunn’s test to compare SCFA profile in semi-stray dogs before and after self-nourished and industrial-nourished diet change. ns: not significant. SCFA, short-chain fatty acids.

**
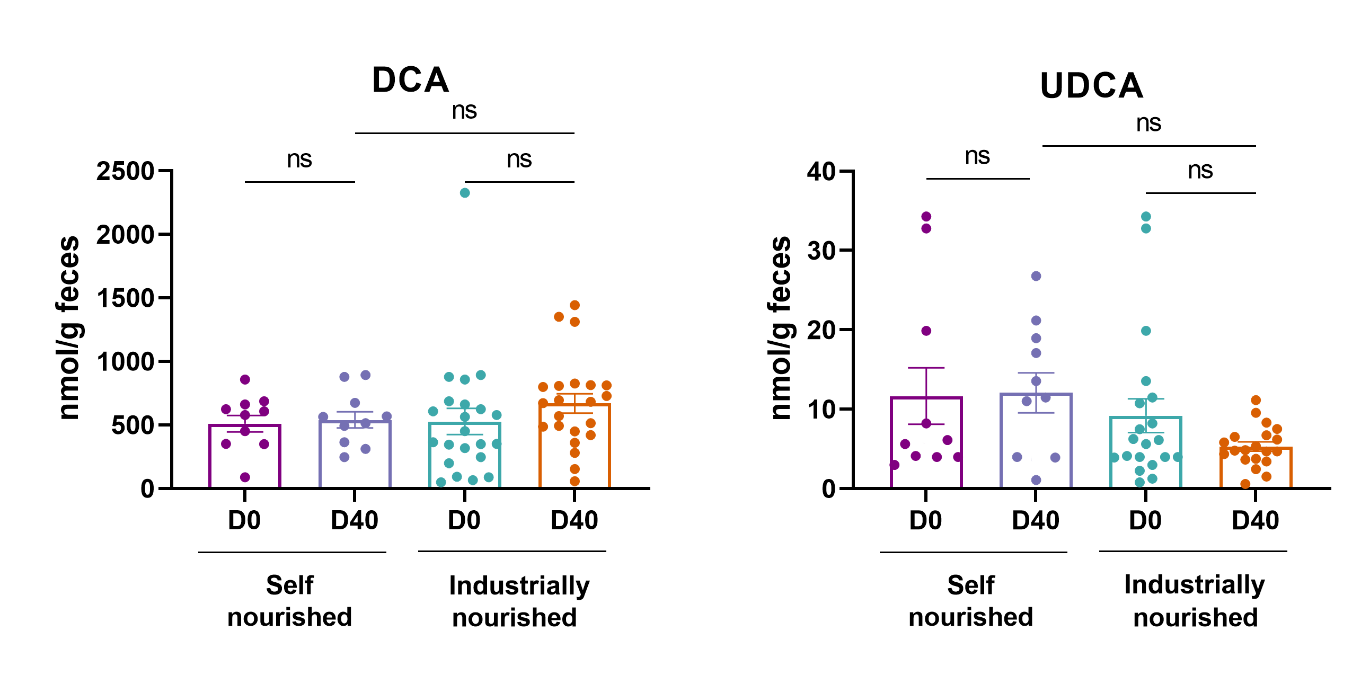
**

**Supplementary Figure 3.** Quantification of bile salts in feces before and after self-nourished and industrial-nourished diet change. Data are presented as means ± SEM. Statistical analyses were performed using Kruskal-Wallis followed by Dunn’s test to compare bile acid profile in semi-stray dogs before and after self-nourished and industrial-nourished diet change. ns: not significant.
